# Management of respiratory failure in immune checkpoint inhibitors-induced overlap syndrome: a case series and review of the literature

**DOI:** 10.1186/s12871-023-02257-z

**Published:** 2023-09-12

**Authors:** John A. Cuenca, Ankit Hanmandlu, Robert Wegner, Joshua Botdorf, Sudhakar Tummala, Cezar A. Iliescu, Joseph L. Nates, Dereddi R. Reddy

**Affiliations:** 1 Department of Critical Care Medicine, Division of Anesthesiology, Critical Care, and Pain, The University of Texas MD Anderson Cancer Center, 1515 Holcombe Blvd., Houston, TX 77030 USA; 2 McGovern School of Medicine, University of Texas, Houston, TX USA; 3 Department of Neuro-oncology, The University of Texas MD Anderson Cancer Center, Houston, TX USA; 4 Department of Cardiology, The University of Texas MD Anderson Cancer Center, Houston, TX USA

**Keywords:** Critical care, Immune checkpoint inhibitor, Immunotherapy, Myasthenia gravis, Myocarditis, Myositis, Oncology

## Abstract

**Background:**

Checkpoint inhibitor-induced overlap syndrome ([OS] myocarditis, and myositis with or without myasthenia gravis) is rare but life-threatening.

**Cases presentation:**

Here we present a case series of four cancer patients that developed OS. High troponinemia raised the concern for myocarditis in all the cases. However, the predominant clinical feature differed among the cases. Two patients showed marked myocarditis with a shorter hospital stay. The other two patients had a prolonged ICU stay due to severe neuromuscular involvement secondary to myositis and myasthenia gravis. Treatment was based on steroids, plasmapheresis, intravenous immunoglobulin, and immunosuppressive biological agents.

**Conclusion:**

The management of respiratory failure is challenging, particularly in those patients with predominant MG. Along with intensive clinical monitoring, bedside respiratory mechanics can guide the decision-making process of selecting a respiratory support method, the timing of elective intubation and extubation.

## Background

The recent advent of immune checkpoint inhibitors (ICI) and immunotherapy have revolutionized the treatment of cancer. ICIs prevent solid and hematological malignancies from evading the natural antitumor response by targeting programmed cell death protein-1 (PD-1) receptor/ligand on T cells and cytotoxic T-lymphocyte-associated antigen 4 (CTLA-4) [1]. Despite these advancements, ICIs can be associated with life-threatening immune-related adverse events such as overlap syndrome (OS) consisting of myasthenia gravis (MG), myositis, and myocarditis [2–4]. The most concerning is ICI-myocarditis, which is associated with a mortality rate of nearly 50% and other related cardiovascular events in up to 46% of cancer patients [5–8]. In contrast, ICI-myositis has a mortality rate of approximately 21%, with half of patients having other severe co-morbidities and prolonged hospital stays [9]. ICI-myocarditis has an incident rate approaching 1%, with concurrent myositis between 30–40% and MG in up to 10% [10]. There is not a clear incidence rate for OS, in part possible due to under recognition and underreporting. However, several cases have been reported in small case series and case reports [2, 3, 7, 8, 11]. Due to the high risk of multiple complications and high in-hospital mortality rates of 60% seen in OS [12], these patients require multidisciplinary management, usually in the intensive care unit (ICU).

Acute respiratory failure is a major risk factor for mortality in critically ill cancer patients [13, 14]. Moreover, a multinational cohort study reported a 90-day mortality rate of 56% among immunocompromised patients with acute hypoxemic respiratory failure [15]. Hence, in patients with OS, the management of respiratory failure is complex, most importantly among patients with MG-predominant clinical picture who develop neuromuscular respiratory failure (NMRF) [2]. Therefore, we present a case series of four patients treated with ICI who developed OS and acute respiratory failure.

## Cases presentations

**Fig. 1:**
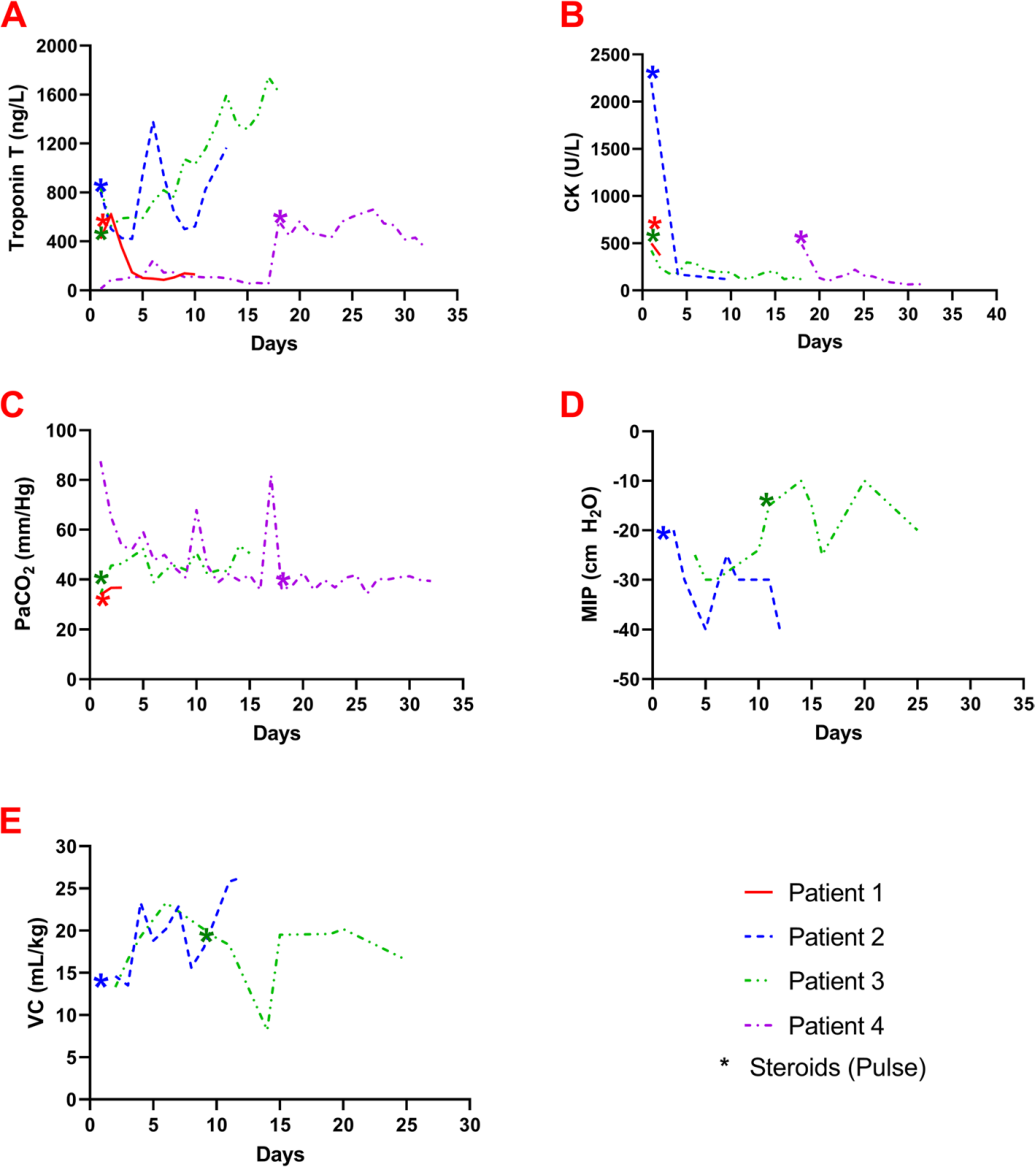
Trends of laboratory parameters and bedside respiratory mechanics by cases. **A** Troponin T. **B** Creatine kinase (CK) **C** Partial pressure of CO2 (PaCO2). **D** maximum inspiratory pressure (MIP). **E** vital capacity (VC)

The Table 1 and Fig. 1 summarize the characteristics of the cases. Written informed consent was obtained.

**Table 1:** Overlap syndrome cases characteristics

	**Case 1**	**Case 2**	**Case 3**	**Case 4**
**Oncologic disease**	Metastatic renal cell carcinoma	Metastatic prostate carcinoma	Metastatic squamous cell carcinoma	Adenocarcinoma of the lung
**BMI**	25.2	31.6	20.2	25.2
**ICI**	Nivolumab/Ipilimumab	Nivolumab/Ipilimumab	Cemiplimab	Nivolumab/Ipilimumab
**Troponin T**, Ref: < 19 ng/L	620 ng/L	1383 ng/L	2,262 ng/L	565 ng/L
**NT Pro-BNP** Ref: < 125 pg/mL	52 pg/ml	2446 pg/ml	827 pg/ml	27,832 pg/mL
**CK**, Ref: < 309 U/L	499 U/L	2,206 U/L	558 U/L	486 U/L
**CK-MB**, Ref: < 10.5 ng/mL	61.2 ng/mL	116 ng/mL	62.6 ng/mL	131 ng/mL
**Aldolase**, Ref: < 7.7 U/L	10.7 U/L	10.2 U/L	43.8 U/L	9.6 U/L
**ECG**	Sinus tachycardia without ischemic changes	New wide QRS morphology	Premature ventricular contractions	Significant for anterolateral ST depression
**Echocardiogram**	No wall motion abnormalities, LVEF 55%	No wall motion abnormalities, LVEF 63%	No wall motion abnormalities, LVEF 64%	Global hypokinesis, LVEF 40%
**EMG**	No neuromuscular junction dysfunction	Axonal and demyelinating motor and sensory peripheral neuropathy	Signs of myositis	Signs of myositis and Guillain–Barre syndrome
**Heart catheterization**	Singe vessel 60% stenosis	Multi vessel disease, 60% stenosis	Multi vessel disease, 60% stenosis	Deferred until stable
**Myocardial biopsy**	Leucocytes infiltration	Leucocytes infiltration	Leucocytes infiltration	Refused
**Muscle biopsy**	Leucocytes infiltration	Leucocytes infiltration	Not performed	Refused

*ECG* Electrocardiogram, *EMG* Electromyography, *ICI* Immune checkpoint inhibitors, *LVEF* Left ventricular ejection fraction, *Ref* Reference normal level, *BMI* Body Mass Index, *NT pro-BNP* N-terminal pro B-Type Natriuretic Peptide

### Case 1

A 62-year-old male with metastatic renal clear cell carcinoma treated with four cycles of nivolumab (PD-1) and ipilimumab (CTLA-4) combination immunotherapy. The patient presented to the emergency department (ED) with dyspnea, tachycardia and weakness. The electrocardiogram (ECG) showed sinus tachycardia without ischemic changes. He had high troponin T, CK, CK-MB, and aldolase. He was placed on high flow oxygen therapy (HFOT), and was admitted to the ICU for suspected ICI-related overlap syndrome and received three pulse-dose steroids (Methylprednisolone 1gr IV per day for 3 days) along with five sessions of plasmapheresis (PLEX). Transthoracic echocardiography showed a 55% left ventricular ejection fraction (LVEF) and no abnormal wall motion. Additional immunosuppression with rituximab was given. Coronary angiography revealed single vessel disease with a 60% stenosis of the left anterior descending artery, deemed non-contributory of the troponin T increase. Electromyography (EMG) showed subtle signs of myopathy with no apparent signs of neuromuscular junction dysfunction. Myocardial and quadriceps biopsies showed leukocyte infiltration consistent with ICI-related myocarditis and myositis. Ten days after admission as the patient displayed clinical improvement, he was discharged home and continued the steroids tapering strategy.

### Case 2

A 76-year-old male with metastatic prostate cancer treated with nivolumab/ipilimumab. The patient had a history of coronary artery disease and a coronary bypass. He presented to the ED with dyspnea, productive cough, and fever for three days. He received empiric antibiotics for potential pneumonia. High troponin T, CK, CK-MB, and aldolase were found. ECG showed a new wide QRS morphology. An echocardiogram showed no signs of wall motion abnormality. However, given his prior cardiovascular history, he received aspirin, atorvastatin and was admitted to the ICU for concerns of overlap syndrome. Three days of pulse-dose steroids and five sessions of PLEX were given. During his ICU course, the patient developed muscle weakness, dysphagia, and dysphonia and required HFOT. Statins were held due to potential myositis. EMG suggested axonal and demyelinating motor and peripheral sensory neuropathy. Multivessel disease and 60% stenosis of the right coronary artery were found on cardiac catheterization. Endomyocardial and muscular biopsies confirmed myocarditis and myositis. As weakness and orthopnea continued despite high-dose steroids and PLEX, treatment with infliximab and rituximab was initiated. After 12 days in the hospital, the patient was discharged home with physical therapy and oral steroids.

### Case 3

A 72-year-old male with metastatic squamous cell carcinoma of the groin presented to the ED, after his third cycle of cemiplimab (PD-1), with worsening lower extremities muscular weakness and dysphagia. The patient was admitted to the ward, and laboratory results found high troponin T, CK, and aldolase, indicating a potential overlap syndrome. The EMG showed signs of myositis. Frequent premature ventricular contractions were reported in the ECG. Paraneoplastic and myasthenia gravis antibodies panel were negative. On day 12 of hospitalization, the patient was found unresponsive after he underwent a percutaneous endoscopic gastrostomy, required emergent intubation with subsequent invasive mechanical ventilation (IMV), and was transferred to the ICU. Despite pulse-dose steroids and PLEX, the troponins remained high. Additional treatment with intravenous immunoglobulin (IVIG), rituximab, and pyridostigmine was given. The cardiac biopsy was consistent with myocarditis. His ICU stay was characterized by profound muscular weakness prompting the need for a tracheostomy. The patient repeatedly failed breathing trials and was unable to liberate from the ventilator due to poor respiratory mechanics. After a month in the ICU, the patient was discharged to a long-term acute care facility.

### Case 4

A 72-year old male with metastatic adenocarcinoma of the lung, treated with nivolumab/ipilimumab admitted to the hospital due to COVID-19 acute respiratory failure. During his hospitalization, oxygen requirements increased, the patient was intubated and admitted to the ICU. The patient developed viral sepsis and required vasopressor support. On day 10 in the COVID ICU, troponin T peaked slowly over a week. A LVEF of 40% and global hypokinesis were found on echocardiogram. ICI-myocarditis and ICI-myositis were suspected due to troponinemia and failure to wean from the ventilator, respectively. The patient received two days of pulse-dose steroids and five sessions of PLEX, and a tracheostomy was placed. EMG showed a mixed pattern of myositis and Guillain–Barre syndrome. Given the critical illness and advanced cancer condition, the patient’s family refused heart catheterization. Thus, based on the use of combination ICI, clinical course, and EMG findings, the medical team determined a presumed diagnosis of OS. After a total of 40 days admitted in the dedicated COVID-19 unit, the patient was transferred to a long-term facility as per family request.

## Discussion and conclusions

**Fig. 2:**
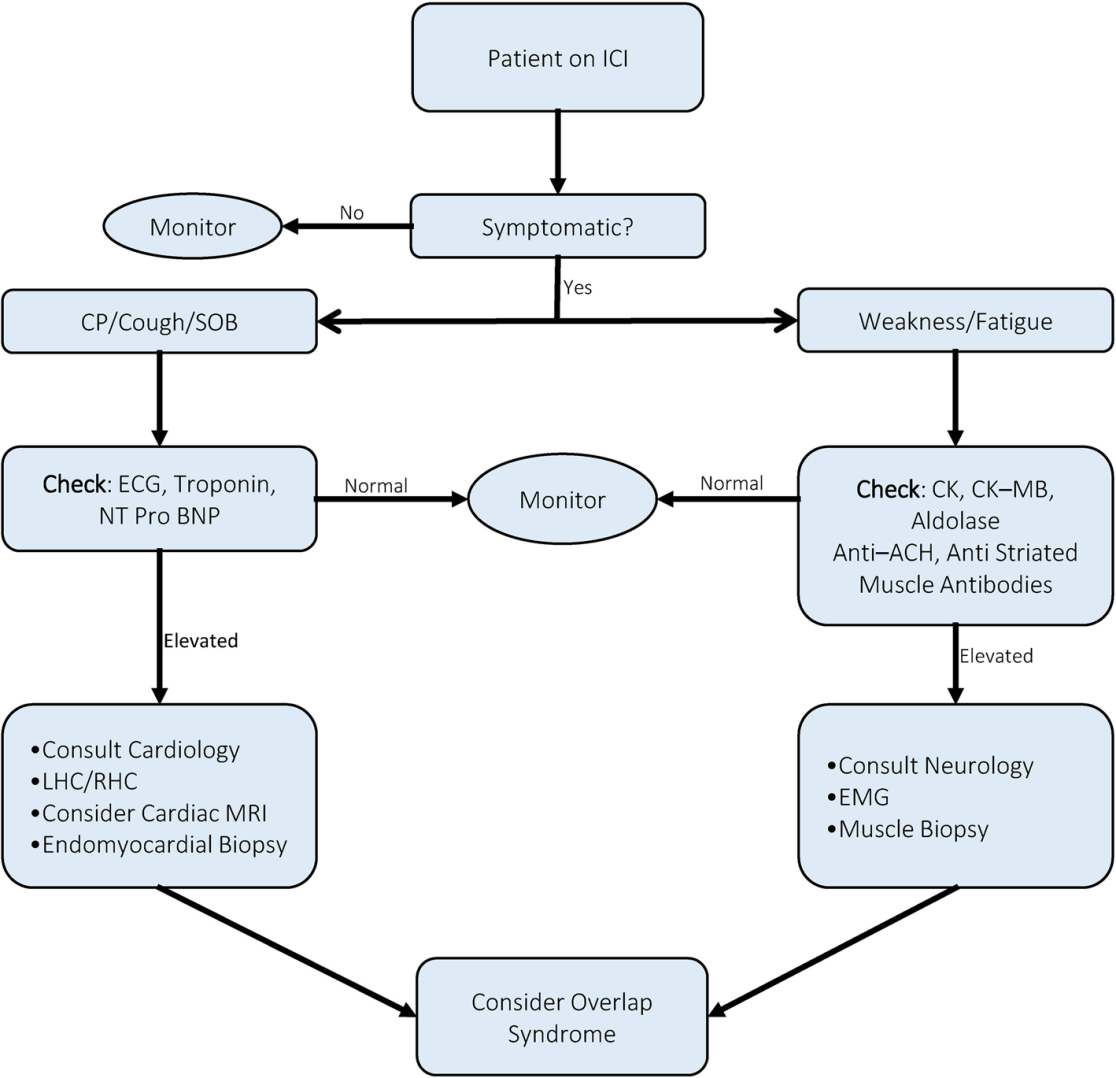
Algorithm for workup of immune checkpoint inhibitors-overlap syndrome. Anti-ACH: Anti-AChR antibodies. CK: Creatine kinase. CK-MB: Creatine kinase-MB. CP: Chest pain. ECG: Electrocardiogram. EMG: Electromyography. ICI: Immune checkpoint inhibitors. LHC: Left heart catheterization. NT Pro BNP: N-terminal pro b-type natriuretic peptide. RHC: Right heart catheterization. SOB: Shortness of breath

We describe a case series of four patients with OS. OS can present with different clinical courses and a predominance of one or more of the components of the syndrome. This raises challenges in the diagnosis of OS; as there is no standard definition or criteria that can be met [1, 6]. Therefore, we propose a stepwise diagnostic approach (Fig. 2). Despite the broad range of clinical syndromes in OS, the therapies are convergent [2]. One of the most challenging aspects is the management of acute respiratory failure; hence, this is the focus of our discussion. This is demonstrated in Case 1 and Case 2, as they were initially admitted for myocarditis related arrhythmias, but had a high risk of impending respiratory failure.

An indicator of potential immune-related adverse event is elevated troponin upon admission [16]. Although troponin I is more clinically specific for ICI-myocarditis, troponin T can also be elevated in patients with concomitant ICI-myositis [17]. Since there was high clinical suspicion for OS, a subsequent diagnostic work-up was performed which included biomarkers as troponin T, CK-MB, CK, and aldolase, ECG to rule out ischemic changes, echocardiogram to rule out myocardial wall dysfunction, left heart catheterization to rule out obstructive coronary artery disease, cardiac biopsy to confirm myocarditis, and EMG and muscle biopsy to confirm myositis and MG. However, due to the often-critical presentation, invasive procedures such as biopsies may be deferred [18]. In such cases, other diagnostic techniques as cardiac MRIs should be considered [19]. Until the understanding of OS increases and a diagnostic criteria is well stablished, clinicians should not defer further investigation in the presence of a single negative test for patients with high degree of suspicion for OS.

In our case series, all patients were admitted into the ICU for ICI-induced OS and received pulse-dose steroids with a subsequent oral steroid tapering strategy, and PLEX. More specifically, Case 1 and 2 were treated empirically with pulse-dose steroids due to high clinical suspicion for OS, Case 3 and 4 were treated once a diagnosis of OS was confirmed. Initial therapy response was measured and subsequently followed by trending troponin T and CK after starting pulse-dose steroids. Since there is no current standardized treatment protocol, all patients were further treated with PLEX, and additional immunosuppression. Of note, electromyography was not able to confirm neuromuscular junction changes consistent with ICI-induced MG. Given our small sample size, this is consistent with other case studies as ICI-induced MG is much more difficult to diagnose with only 57% of patients having clear electrodiagnostic features of MG (41%) or MG and myopathy (16%) [20]. Although, OS has a high mortality rate approaching 60% [12]; all our patients were discharged home or to long-term facilities. This could be due to early recognition and our aggressive treatment approach.

To identify efficacious treatment approaches towards ICI-induced MG, it is important to characterize the differences between ICI-induced MG and idiopathic MG (iMG). Cases of iMG typically have an insidious clinical course, taking 2–3 years to develop symptoms of NMRF requiring support with non-invasive ventilation (NIV) or IMV [21, 22]. However, patients with ICI-induced MG can progress to NMRF in a median time of 7 days [20], as demonstrated in Case 3. Therefore, ICI-induced MG is associated with worse clinical outcomes than with iMG, including a higher risk of respiratory paralysis and death [23]. ICI-MG is also increasingly difficult to diagnose, since there are lower positivity rates in electrodiagnostic testing and lower seropositivity of anti-AChR antibodies than there is in iMG [1]. These differences in presentation and diagnosis could also lead to differences in management. While corticosteroids are a standard of care (in conjunction with IVIG and PLEX) and lead to more favorable outcomes of ICI-induced MG in OS [20], corticosteroid use can increase the progression of respiratory failure in iMG [24].

Bedside respiratory mechanics test and arterial blood gas can be used as prognostic indicators in a variety of diseases, but their use in NMRF and OS is poorly understood. Findings in early NMRF include hyperventilation with rapid/shallow breaths resulting in hypocapnia that progress in late NMRF to hypoventilation with hypercapnia [25, 26]. When PaCO_2_ reaches between 40–45 mmHg and pH < 7.35, this indicates respiratory failure. However, bedside respiratory mechanics tests have been assessed to predict the need for respiratory support. In a study by Seneviratne and colleagues, arterial gases had poor predictive value of the duration and outcome of NIV or IMV in myasthenic crisis [27]. Contrarily, a systematic review showed that patients with a maximum expiratory pressure (MEP) > 40 cm H_2_O, vital capacity (VC) > 20 mL/kg or maximum inspiratory pressure (MIP) < -40 cm H_2_O typically do not need mechanical ventilation [28]. Due to the fluctuating clinical course of NMRF in OS, symptom severity is not a reliable predictor of improvement and stability. Nonetheless, general principles should be used to treat the underlying neuromuscular disorder, use NIV in eligible patients, and IMV when necessary. While there is not much data regarding treatment of NMRF in OS, we can use this case series and myasthenic crisis as a reference that warrants further investigation.

Bilevel positive airway pressure (BiPAP) is preferred in myasthenic crisis because it can model natural respiratory mechanics. Ventilatory failure due to respiratory fatigue and dysfunction are the predominant mechanism of NMRF [25]. BiPAP allows for modifiable and continuous positive pressures that decreases the risk for both atelectasis and upper airway collapse [27]. The benefits of BiPAP are significant, as 20% of patients in myasthenic crisis can be supported solely by NIV [29]. Predictors of NIV success include an Acute Physiologic Assessment and Chronic Health Evaluation II score < 6, bicarbonate < 30 mEq/L, and absence of overt hypercapnia (PaCO2 > 50 mmHg strongly correlated with failure; *p* < 0.01) [30, 31]. Patients managed initially with NIV prior to intubation require a shorter duration of ventilator support in comparison to patients only managed with IMV (4 vs. 9 days) [27]. Furthermore, prolonged IMV increases the risk of atelectasis, lowers MEP and is a frequent cause of longer ICU stays due to ventilator-associated pneumonia and other systemic complications [27]. A BiPAP trial before established hypercapnia can prevent prolonged ventilation and intubation [27].

If NIV fails to improve the patient’s respiratory status, intubation will need to occur without delay. Nearly 66%-90% of patients in myasthenic crisis require IMV at the emergency department or after admission into the ICU [32, 33]. Some subjective indications for intubation in NMRF are decreased levels of consciousness, diaphragmatic fatigue, bilateral facial and bulbar weakness (dysarthria, dysphagia, impaired gag reflex, staccato speech) [34]. Furthermore, hemodynamic instability, dysautonomia, and a deteriorating clinical course are objective indications that warrant intubation [34]. After successful intubation, patient’s ventilator settings and the degree of respiratory support is largely patient dependent [35]. Of note, neuromuscular blockers should be used cautiously with ICI-induced MG patients. This is because the anti-ACh-R antibodies reduce the amount of functional ACh-R available for neurotransmission. Hence, depolarizing agents become less potent, while non-depolarizing agents increase their potency [36].

Predictors for prolonged ventilation in NMRF are integral in guiding the proper timing of a tracheostomy. Thomas and colleagues used a pre-intubation bicarbonate ≥ 30 mEq/L, peak VC on days 1–6 after intubation of < 25 mL/kg, and age > 50 years old to assess for patients that required prolonged ventilation in myasthenic crisis beyond 2 weeks [32]. Some studies have shown that early tracheostomy was beneficial and resulted in decreased incidence of ventilator-associated pneumonia [37], decreased use of sedation [38], earlier ICU discharge [39], and lower mortality [39]. However, other studies found no difference in length of stay [40], or mortality [41]. These conflicting results call for large clinical trials to address this clinical dilemma.

After adequate respiratory and clinical improvement, weaning from IMV can occur. Generally, patients should have: few secretions, an adequate cough reflex, and tolerate minimal pressure support for four hours without showing symptoms of respiratory fatigue [25]. A VC greater than 10–15 mL/kg for at least 4 h was necessary before extubation could be considered [35, 42, 43]. In addition to these objective measurements, evaluating for improvement in the strength and tone of neck flexors and accessory respiratory muscles are important [43]. After extubation, patients should be transitioned to NIV [29]. The best predictor of extubation success is an improvement in the MEP [29]. Contrarily, extubation failure is most commonly associated with weak cough, inadequate airway clearance, older age, atelectasis, pneumonia, acidosis, decreased VC, and the need for NIV [31, 44, 45]. Hence, in patients with clinical suspicion of difficulty extubating, a trial of extubation over a Cook catheter can be performed [46]. Despite this, re-intubation still occurs almost 25% of the time [44, 45]. If re-intubation is imminent, tracheostomy placement can also be considered [45, 47].

The present report has limitations inherent to the study setting and design that need to be considered. First, this is a single-center, retrospective, small case series. Second, it was conducted in a Comprehensive Cancer Center with high volumes of complex cases, which introduce referral bias by including a more severe population. Third, the high acuity of the cases presented in this series could be also associated to the combination ICI therapy that three patients received, which has been previously reported as having higher immune-related adverse events mortality rates [10]. Further reports with higher volume of patients could better describe the association between physiological parameters, radiological findings, specific ventilation techniques with disease progression and outcomes.

As the use of checkpoint inhibitors continue to expand, the incidence of rare side effects such as OS will also increase. Despite the difficulties conducting research in critically ill cancer patients [48], large cohorts studies are required to understand the characteristics and outcomes of patients with OS.

Although myocarditis occurs in less than 1% of patients receiving ICIs, once it presents, the risk of developing associated myositis and MG is 40% and 10%, respectively. Therefore, in any patient with ICI-related myocarditis, OS should be suspected and thoroughly investigated. OS is clinically diverse and potentially fatal and requires a multidisciplinary assessment. While there is no consensus, treatment is based on steroids, plasmapheresis, IVIG, and immunosuppressive biological agents. The management of respiratory failure is challenging, particularly in those patients with predominant MG. Along with intensive clinical monitoring, bedside respiratory mechanics can guide the decision-making process of selecting a respiratory support method, the timing of elective intubation and extubation. Larger cohort studies are needed to fully understand the characteristics and outcomes of Overlap syndrome.

## Data Availability

All data analysed during this study are included in this published article.

## Abbreviations

BiPAP: Bilevel positive airway pressure
CTLA-4: Cytotoxic T-lymphocyte-associated antigen 4
ICI: Immune checkpoint inhibitors
ECG: Electrocardiogram
ED: Emergency department
EMG: Electromyography
HFOT: High flow oxygen therapy
ICU: Intensive care unit
iMG: Idiopathic MG
IMV: Invasive mechanical ventilation
IVIG: Intravenous immunoglobulin
LVEF: Left ventricular ejection fraction
MEP: Maximum expiratory pressure
MG: Myasthenia gravis
MIP: Maximum inspiratory pressure
NIV: Non-invasive ventilation
NMRF: Neuromuscular respiratory failure
OS: Overlap syndrome
PD-1: Programmed cell death protein-1
PLEX: Plasmapheresis
VC: Vital capacity

## References

[1] Pathak R, Katel A, Massarelli E, Villaflor VM, Sun V, Salgia R (2021). Immune checkpoint inhibitor-induced myocarditis with myositis/myasthenia gravis overlap syndrome: a systematic review of cases. Oncologist.

[2] Lipe DN, Galvis-Carvajal E, Rajha E, Wechsler AH, Gaeta S (2021). Immune checkpoint inhibitor-associated myasthenia gravis, myositis, and myocarditis overlap syndrome. Am J Emerg Med.

[3] Jeyakumar N, Etchegaray M, Henry J, Lelenwa L, Zhao B, Segura A (2020). The terrible triad of checkpoint inhibition: a case report of myasthenia gravis, myocarditis, and myositis induced by cemiplimab in a patient with metastatic cutaneous squamous cell carcinoma. Case Reports in Immunol.

[4] Cuenca JA, Laserna A, Reyes MP, Nates JL, Botz GH (2020). Critical care admission of an HIV patient with diabetic ketoacidosis secondary to pembrolizumab. Case Rep Crit Care.

[5] Todo M, Kaneko G, Shirotake S, Shimada Y, Nakano S, Okabe T (2020). Pembrolizumab-induced myasthenia gravis with myositis and presumable myocarditis in a patient with bladder cancer. IJU Case Reports.

[6] Xing Q, Zhang ZW, Lin QH, Shen LH, Wang PM, Zhang S (2020). Myositis-myasthenia gravis overlap syndrome complicated with myasthenia crisis and myocarditis associated with anti-programmed cell death-1 (sintilimab) therapy for lung adenocarcinoma. Ann Transl Med.

[7] Rota E, Varese P, Agosti S, Celli L, Ghiglione E, Pappalardo I (2019). Concomitant myasthenia gravis, myositis, myocarditis and polyneuropathy, induced by immune-checkpoint inhibitors: a life–threatening continuum of neuromuscular and cardiac toxicity. eNeurologicalSci.

[8] Fazel M, Jedlowski PM (2019). Severe myositis, myocarditis, and myasthenia gravis with elevated anti-striated muscle antibody following single dose of ipilimumab-nivolumab therapy in a patient with metastatic melanoma. Case Reports Immunol.

[9] Anquetil C, Salem J-E, Lebrun-Vignes B, Johnson DB, Mammen AL, Stenzel W (2018). Immune checkpoint inhibitor-associated myositis. Circulation.

[10] Moslehi JJ, Salem J-E, Sosman JA, Lebrun-Vignes B, Johnson DB (2018). Increased reporting of fatal immune checkpoint inhibitor-associated myocarditis. Lancet.

[11] Shirai T, Kiniwa Y, Sato R, Sano T, Nakamura K, Mikoshiba Y (2019). Presence of antibodies to striated muscle and acetylcholine receptor in association with occurrence of myasthenia gravis with myositis and myocarditis in a patient with melanoma treated with an anti–programmed death 1 antibody. Eur J Cancer.

[12] Johnson DB, Manouchehri A, Haugh AM, Quach HT, Balko JM, Lebrun-Vignes B (2019). Neurologic toxicity associated with immune checkpoint inhibitors: a pharmacovigilance study. J Immunother Cancer.

[13] Manjappachar NK, Cuenca JA, Ramírez CM, Hernandez M, Martin P, Reyes MP (2022). Outcomes and predictors of 28-day mortality in patients with hematologic malignancies and septic shock defined by sepsis-3 criteria. J Natl Compr Canc Netw.

[14] Cuenca JA, Manjappachar NK, Ramírez CM, Hernandez M, Martin P, Gutierrez C (2022). Outcomes and predictors of 28-day mortality in patients with solid tumors and septic shock defined by third international consensus definitions for sepsis and septic shock criteria. Chest.

[15] Azoulay E, Pickkers P, Soares M, Perner A, Rello J, Bauer PR (2017). Acute hypoxemic respiratory failure in immunocompromised patients: the Efraim multinational prospective cohort study. Intensive Care Med.

[16] Delombaerde D, Vervloet D, Franssen C, Croes L, Gremonprez F, Prenen H (2021). Clinical implications of isolated troponinemia following immune checkpoint inhibitor therapy. ESMO Open.

[17] Bonaca MP, Olenchock BA, Salem J-E, Wiviott SD, Ederhy S, Cohen A (2019). Myocarditis in the setting of cancer therapeutics. Circulation.

[18] van der Boon RMA, den Dekker WK, Meuwese CL, Lorusso R, von der Thüsen JH, Constantinescu AC (2021). Safety of endomyocardial biopsy in new-onset acute heart failure requiring veno-arterial extracorporeal membrane oxygenation. Circulation.

[19] Arcari L, Tini G, Camastra G, Ciolina F, De Santis D, Russo D (2022). Cardiac magnetic resonance imaging in immune check-point inhibitor myocarditis: a systematic review. J Imaging.

[20] Safa H, Johnson DH, Trinh VA, Rodgers TE, Lin H, Suarez-Almazor ME (2019). Immune checkpoint inhibitor related myasthenia gravis: single center experience and systematic review of the literature. J Immunother Cancer.

[21] Mantegazza R, Beghi E, Pareyson D, Antozzi C, Peluchetti D, Sghirlanzoni A (1990). A multicentre follow-up study of 1152 patients with myasthenia gravis in Italy. J Neurol.

[22] Grob D, Brunner N, Namba T, Pagala M (2008). Lifetime course of myasthenia gravis. Muscle Nerve.

[23] Groth A, Vrugt B, Brock M, Speich R, Ulrich S, Huber LC (2014). Inflammatory cytokines in pulmonary hypertension. Respir Res.

[24] Bae JS, Go SM, Kim BJ (2006). Clinical predictors of steroid-induced exacerbation in myasthenia gravis. J Clin Neurosci.

[25] Mehta S (2006). Neuromuscular disease causing acute respiratory failure. Respir Care.

[26] Singh TD, Wijdicks EFM (2021). Neuromuscular respiratory failure. Neurol Clin.

[27] Seneviratne J, Mandrekar J, Wijdicks EFM, Rabinstein AA (2008). Noninvasive ventilation in myasthenic crisis. Arch Neurol.

[28] Thieben MJ, Blacker DJ, Liu PY, Harper CM, Wijdicks EFM (2005). Pulmonary function tests and blood gases in worsening myasthenia gravis. Muscle Nerve.

[29] Wendell LC, Levine JM (2011). Myasthenic crisis. Neurohospitalist.

[30] Rabinstein A, Wijdicks EFM (2002). BiPAP in acute respiratory failure due to myasthenic crisis may prevent intubation. Neurology.

[31] Wu J-Y, Kuo P-H, Fan P-C, Wu H-D, Shih F-Y, Yang P-C (2009). The role of non-invasive ventilation and factors predicting extubation outcome in myasthenic crisis. Neurocrit Care.

[32] Thomas CE, Mayer SA, Gungor Y, Swarup R, Webster EA, Chang I (1997). Myasthenic crisis: clinical features, mortality, complications, and risk factors for prolonged intubation. Neurology.

[33] O’Riordan JI, Miller DH, Mottershead JP, Hirsch NP, Howard RS (1998). The management and outcome of patients with myasthenia gravis treated acutely in a neurological intensive care unit. Eur J Neurol.

[34] Rabinstein AA, Wijdicks EFM (2003). Warning signs of imminent respiratory failure in neurological patients. Semin Neurol.

[35] Kirmani JF, Yahia AM, Qureshi AI (2004). Myasthenic crisis. Curr Treat Options Neurol.

[36] Baraka A (1992). Anaesthesia and myasthenia gravis. Can J Anaesth.

[37] Siempos II, Ntaidou TK, Filippidis FT, Choi AMK (2015). Effect of early versus late or no tracheostomy on mortality and pneumonia of critically ill patients receiving mechanical ventilation: a systematic review and meta-analysis. Lancet Respir Med.

[38] Szakmany T, Russell P, Wilkes AR, Hall JE (2015). Effect of early tracheostomy on resource utilization and clinical outcomes in critically ill patients: meta-analysis of randomized controlled trials. Br J Anaesth.

[39] Andriolo BN, Andriolo RB, Saconato H, Atallah ÁN, Valente O. Early versus late tracheostomy for critically ill patients. Cochrane Database Syst Rev. 2015;2017. 10.1002/14651858.CD007271.pub3. 10.1002/14651858.CD007271.pub3 PMC6517297 25581416

[40] Terragni PP, Antonelli M, Fumagalli R, Faggiano C, Berardino M, Pallavicini FB (2010). Early vs late tracheotomy for prevention of pneumonia in mechanically ventilated adult ICU patients. JAMA.

[41] Young D, Harrison DA, Cuthbertson BH, Rowan K, TracManCollaborators for the (2013). Effect of early vs late tracheostomy placement on survival in patients receiving mechanical ventilation. JAMA.

[42] Rieder P, Louis M, Jolliet P, Chevrolet JC (1995). The repeated measurement of vital capacity is a poor predictor of the need for mechanical ventilation in myasthenia gravis. Intensive Care Med.

[43] Meriggioli MN (2009). Myasthenia gravis: Immunopathogenesis, diagnosis, and management. Continuum.

[44] Rabinstein AA, Mueller-Kronast N (2005). Risk of extubation failure in patients with myasthenic crisis. Neurocrit Care.

[45] Seneviratne J, Mandrekar J, Wijdicks EFM, Rabinstein AA (2008). Predictors of extubation failure in myasthenic crisis. Arch Neurol.

[46] Moyers G, McDougle L (2002). Use of the cook airway exchange catheter in “bridging” the potentially difficult extubation: a case report. AANA J.

[47] Kulkarni A, Agarwal V (2008). Extubation failure in intensive care unit: predictors and management. Indian J Crit Care Med.

[48] Reyes MP, Cuenca JA, Juliana H, Martin PR, Villalobos DHD, Nates JL (2022). Tribulations of conducting critically ill cancer patients research: lessons from a failed septic shock trial and Murphy’s law. Med Intensiva.

